# The Occurrence of Microplastics and the Formation of Biofilms by Pathogenic and Opportunistic Bacteria as Threats in Aquaculture

**DOI:** 10.3390/ijerph19138137

**Published:** 2022-07-02

**Authors:** Paulina Cholewińska, Hanna Moniuszko, Konrad Wojnarowski, Przemysław Pokorny, Natalia Szeligowska, Wojciech Dobicki, Ryszard Polechoński, Wanda Górniak

**Affiliations:** 1 Institute of Animal Breeding, Wroclaw University of Environmental and Life Sciences, 51-630 Wroclaw, Poland; paulina.cholewinska@upwr.edu.pl (P.C.); przemyslaw.pokorny@upwr.edu.pl (P.P.); natalia.szeligowska@upwr.edu.pl (N.S.); wojciech.dobicki@upwr.edu.pl (W.D.); ryszard.polechonski@upwr.edu.pl (R.P.); 2 Section of Applied Entomology, Department of Plant Protection, Institute of Horticultural Sciences, Warsaw University of Life Sciences–SGGW, Nowoursynowska 159, 02-776 Warsaw, Poland; hanna_moniuszko@sggw.edu.pl; 3 Chair for Fish Diseases and Fisheries Biology, Ludwig-Maximilians-University of Munich, 80539 Munich, Germany; 4 Department of Automotive Engineering, Mechanical Faculty, Wrocław University of Science and Technology, Na Grobli 13, 50-421 Wroclaw, Poland; wanda.gorniak@pwr.edu.pl

**Keywords:** microplastics, aquaculture, biofilm, microbiome, pathogenic bacteria, fish, human

## Abstract

Aquaculture is the most rapidly growing branch of animal production. The efficiency and quality of the produced food depends on sustainable management, water quality, feed prices and the incidence of diseases. Micro- (MP < 5 mm) and nanoplastic (NP < 1000 nm) particles are among the current factors causing serious water pollution. This substance comes solely from products manufactured by humans. MP particles migrate from the terrestrial to the aquatic environment and adversely affect, especially, the health of animals and humans by being a favorable habitat and vector for microbial pathogens and opportunists. More than 30 taxa of pathogens of humans, aquacutural animals and plants, along with opportunistic bacteria, have been detected in plastic-covering biofilm to date. The mobility and durability of the substance, combined with the relatively closed conditions in aquacultural habitats and pathogens’ affinity to the material, make plastic particles a microbiological medium threatening the industry of aquaculture. For this reason, in addition to the fact of plastic accumulation in living organisms, urgent measures should be taken to reduce its influx into the environment. The phenomenon and its implications are related to the concept of one health, wherein the environment, animals and humans affect each other’s fitness.

## 1. Introduction

In over 25 years, the branch of aquaculture has developed to supply circa half of the fish demand worldwide, with Asia being the largest market. About 90% of the global demand for farmed fish and crustaceans—mainly tilapia and shrimp—is supplied from this area [1]. It is the fastest growing food production sector in the world, with an approximately 60% share of freshwater fish [2].

Aquaculture depends on sustainable management, adequate water quality, feed prices and the avoidance of diseases [3,4]. The latter has been recently connected with the problem of microbiome in farmed animals, including fish and shellfish. Attention has been paid to the crucial role that the microbiome plays in animal health and productivity [2,5]. Water is one of the most important habitats for microorganisms on Earth; at the same time, it is the main way that they spread in the environment [6,7,8]. The most numerous water-borne microorganisms are bacteria, which are also the most diverse compared to other groups. So, the microbiologically diverse habitat of fish may affect them, both positively (gains improvement, good health and fitness) negatively by increasing mortality [8,9]. Microbiome composition in fresh and salty waters highly depends on physico-chemical conditions, such as temperature, salinity, electrical conductivity, mineral composition and, as has been recently confirmed, the occurrence of microplastic particles [10,11,12,13].

Plastic was invented in 1907, and has since become commonly used, ranging from the food to the medical industries. The material is a polymeric compound, obtained as a result of addition polymerization or polycondensation reactions. More than half is currently being produced in Asia, with China providing circa 30% of the world’s production. In 2020, around 367 million tons of this material were produced, and the trade balance was EUR 16 billion [14,15,16].

Microplastics (MP < 5 mm) and nanoplastics (NPP < 1000 nm) gained attention in the aquacultural section due to their wide occurrence in the environment combined with difficulties in their removal from waters. Plastic, including microplastic, constitutes 95% of marine litter, and an equally important fact is that the material decomposes very slowly or not at all [17,18,19]. As a consequence, it has accumulative potential in trophic chains. The research conducted by Chen et al. [20] proved the bioaccumulation of bisphenol A (BPA) in the viscera, gills, head and muscular tissue of *Danio rerio*, after only one-day exposure (BPA concentration of 0.78 ± 0.09 μg/L) under laboratory conditions. The greatest amount of BPA was recorded in the viscera (85 ug/g), while the lowest in muscles (3 ug/g). The study also revealed that exposure to combined (NPP with BPA) factors resulted in a 2.2–2.6-fold increase in BPA uptake by the head and bowels, compared to contact only with BPA or NPP. Additionally, in tested fish, the regulation of the myelin basic protein (MBP) gene has changed, which affects the central nervous system, and the activity of acetylcholinesterase (AChE, a neurotoxicity biomarker) is inhibited. The pace of plastic accumulation in living organisms varies greatly and depends on the presence of city agglomerations, sewage treatment plants, type of reservoir, development of shores and hydrodynamics. Finally, bacterial biofilms covering plastic particles can increase the risk of the occurrence of farmed animals’ diseases, thus negatively influencing food production [13,16,21,22,23].

This article reviews our current understanding of the presence of microplastics and the associated formation of bacterial biofilm as a threat to organisms kept in aquaculture. The literature was browsed in the period of 25 February 2022–20 March 2022 in Google Scholar, Web of Science and Scopus databases using the following keywords: microplastic, aquaculture, microbiome, biofilm, fish, pathogens, carp, Atlantic salmon, rainbow trout, bream, tilapia, and shrimp. The analysis of the available literature led to the inclusion of 145 research papers published from 1977 to 2022 in the present review paper.

## 2. Occurrence of Microplastics in Aquaculture

Microplastics represent a very heterogeneous group in the environment due to their shape, size, chemical composition or specific density. They can be divided into two types, primary and secondary. Primary MPs include the particles of plastic that are produced and released into the environment, while secondary MPs are produced by the shredding of larger particles. Most MPs, about 80%, enter the waters from land. For example, in 2020, the EU cosmetic industry produced 4459 tons of MP. An additional problem is wastewater entering the oceans. This is prohibited in most countries; however, only 15–20% of wastewater is treated worldwide, while treatment plants are able to remove about 90% of MPs. Moreover, plastic enters the aquatic environment from agrocenoses. In agriculture, waste residues can be used to fertilize soils where they can enter water with surface runoff after heavy rainfalls [24].

**Figure 1:**
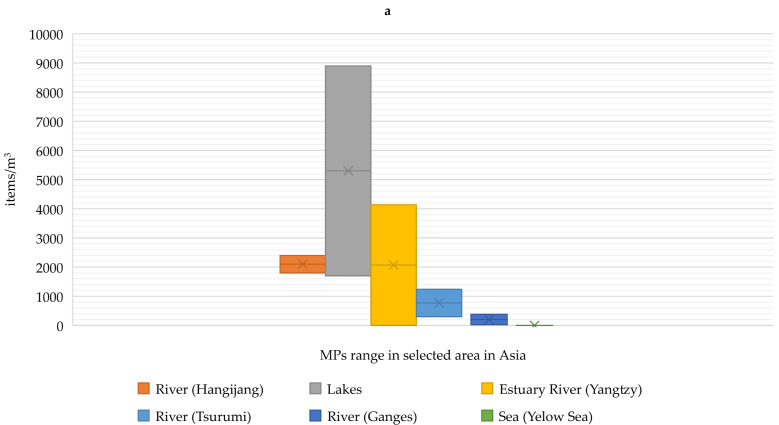

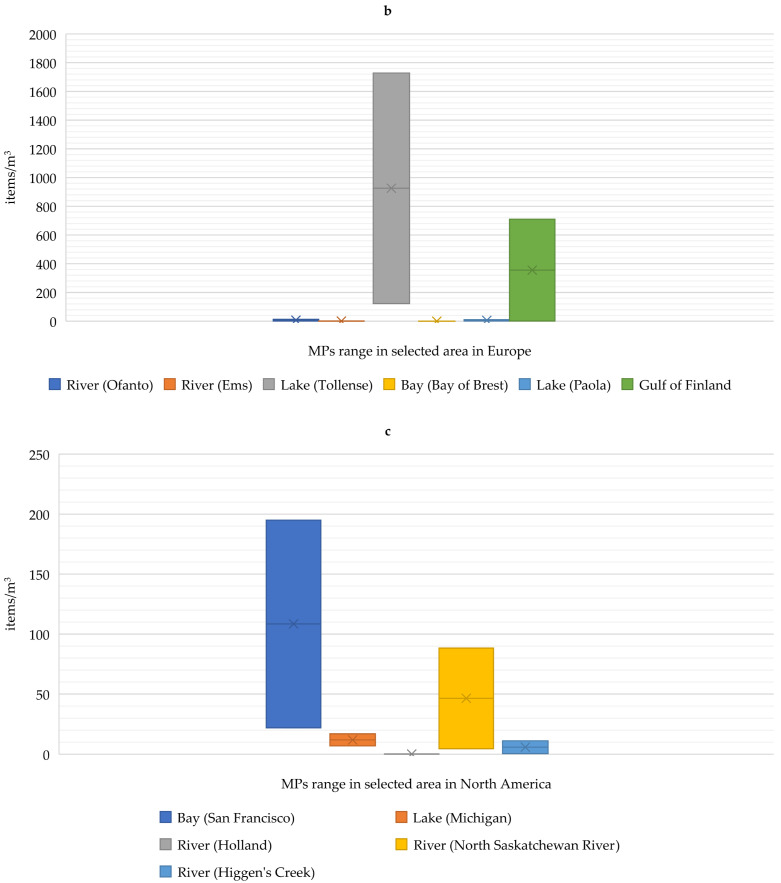
MP range (<5 mm) at selected locations ((**a**)—Asia; (**b**)—Europe; (**c**)—North America) [25,26,27,28,29,30,31,32,33,34,35,36,37,38,39,40,41,42,43].

MPs have been detected in oceans around the world; however, the highest concentrations have been observed near industrialized or metropolitan areas (Figure 1). High concentrations have also been observed in enclosed or semi-enclosed seas, such as the Mediterranean Sea. Estimates assume that 20 Mt/year of MPs enters the oceans, and this amount is expected to triple by 2030 [24,25]. In the study by Deng et al. [26] where microplastic levels were investigated in industrialized areas (Shaoxing textile industry, China), the occurrence of 2.1–71.0 pcs/L in surface water and 16.7–1323.3 pcs/kg in bottom sediments was reported. In the study by Luo et al. [27] on different types of water bodies in China, it was shown that the highest number of MPs was found in freshwater bodies: 1.8–2.4 pcs/L, where the level in estuaries and coastal areas of the East China Sea (Yangtze River delta area) was at 0.9 pcs/L.

**Figure 2:**
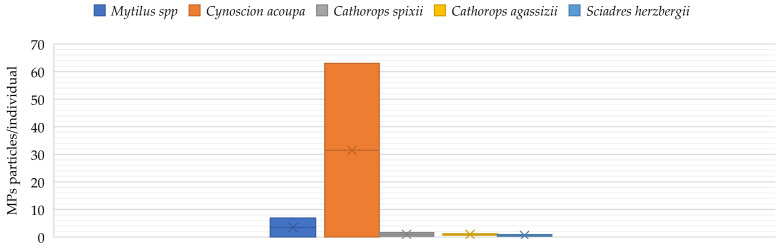
MP range (<5 mm) in selected marine species [45,46,48,49].

MPs have significant, but detrimental, effects on aquatic biota, mainly at lower trophic levels. Numerous publications document their presence in aquatic organisms (Figure 2). [25]. The study by Lusher et al. [44] was the first to demonstrate the occurrence of MPs in the gastrointestinal tract in cetaceans. Bråte et al. [45] in a study on *Mytilus* spp. showed that mussels occurring along the Norwegian coastline contained MPs, except for individuals inhabiting the west coast. The highest levels of MP contamination were observed in individuals in Barent’s Sea and around large agglomerations (0.97–1.5 particles ind −1). This came mainly from nets used for fishing (nylon). In a study conducted during the same period by Ferrieira et al. [46] on the economically important estuarine apex predator *Cynoscion acoupa* (n = 552), it was shown that MPs were detected in more than half of the fish examined. Additionally, it was noted that MPs accumulated more frequently in adults than juveniles. In comparison, in a study conducted by Bellas et al. [47] on demersal fish living off the Atlantic and Mediterranean coasts (*Scyliorhinus canicula*, *Merluccius merluccius* and *Mullus barbatus*), the percentage of fish with MPs in the body was 17.3%. The highest level was detected in *M. barbatus* with 18.8%. The size of MP detected in fish was in the ranged of 0.38–3.1 mm. Similar results were obtained in a study by Possatto et al. [48] on *Cathorops spixii* (N = 60), *C. agassizii* (N = 60) and *Sciadres herzbergii* (N = 62) (Atlantic estuary on the south-eastern side of Brazil) showed that between 18 and 33% of individuals had MP particles in their digestive tract. However, in a study by Nadal et al. [49], where the research was conducted on semi-pelagic fish from the Mediterranean Sea (Belara), MPs were found in the digestive system in 68% of the individuals tested. The ingestion rate was approximately 3.75 MPs particles/article, and their size ranged from 1 nm to 5 mm. In contrast, the study by Rummel et al. [50] in the North Sea and Baltic Sea detected MPs in 5.5% of all groundfish and pelagic fish examined, with 75% of the particles being MPs (<5 mm). Additionally, it was shown that 40% of the MP particles examined were composed of polyethylene. In a study by Choi et al. [51], the Mediterranean clams *Mytilus galloprovincialis* were exposed to MPs (0.5 μg/L and 100 mg/L) for 4 days. The results showed the accumulation of MPs in the intestinal organs. In addition, the study demonstrated the effect of the presence of MPs in the gastrointestinal tract of mussels on increasing the risk of necrosis, DNA damage or acetylcholinesterase (AChE) activity. In contrast, in the Spanjer et al. [52] study on juvenile Chinook salmon, the fish were able to remove approximately 94% of the MP within 10 days. This study also showed no effect of MPs on the average gastrointestinal weight of the test individuals, suggesting that there was no change in digestive rate. The authors also emphasize that there is a need for further research to understand if repeated exposure to MPs can affect food absorption, including fish growth. Additionally, a study by Kim et al. [53] on American brine shrimp (*Artemia franciscana*) exposed to MPs (polypropylene and polyethylene terephthalon-PE) showed increased mortality in the PE-exposed group compared to controls or individuals exposed to polypropylene. In contrast, polypropylene caused significant intestinal damage compared to the other groups. It was also found in the studies of Au et al. [54] and Besseling et al. [55] to have negative effects on food absorption, body weight, growth and reproductive performance. The Au et al. [54] study also showed that polypropylene fibers were significantly more toxic to *Hyalella azteca* than polyethylene fibers. In contrast, a study by Besseling et al. [55] showed that MPs increased the bioaccumulation of polychlorinated biphenyls (PCBs) in the body of *Arenicola marina*, which have the ability to modulate endocrine activity and contribute to the development of reproductive anomalies [55,56]. A study by Lorite et al. [57] showed that, in addition to the surface structure of the material, the coating layers consisting of proteins and other biomolecules around nanoparticles in biological fluids, such as serum and cytoplasm, which affect the physicochemical interactions of nanomaterials with cells, are equally important.

## 3. Biofilm Formation

Many environments and organisms are open systems colonized by microorganisms, most of which form a biofilm. The majority of them are symbiotic organisms; however, the opportunistic are also part of them [58,59]. Understanding biofilm formation and function contributes to reducing, among other things, the incidence of infections; adverse changes associated with biofilm formation are associated with the spread of infections that are difficult to treat, in both animals and humans [60,61,62]. A biofilm may consist of one or more microorganisms surrounded by an extracellular layer of polysaccharides, etc. [63,64].

Biofilm formation on synthetic polymers, such as polyethylenes (LDPE, HDPE, LLDPE) and polyvinyl chloride, due to the composition and amounts of additives, such as pro-oxidants and starch, affect the species composition of biofilm microorganisms. In addition, microbial colonization can also alter the properties of microplastics by affecting the carbonyl indices as well as the number of double bonds [65]. In a study by Rogers et al. [66], significantly higher numbers of bacteria were detected on plastics than on copper surfaces. The tested *Legionella pneumophilia* was present on the polyvinyl chloride surface even at 50 °C, where it was no longer detected on copper. The tested surfaces of PVCc (chlorinated polyvinyl chloride), PVCu (unplasticized polyvinyl chloride) or polyethylene showed significantly higher bacterial counts compared to stainless steel or mild steel (2.24–7.75 × 104 on synthetic polymers, 1.03–2.13 × 10^4^ on metals). Research by Parrish and Fahrenfeld [67] also suggests that both material type and size affect biofilm formation and composition. In addition to different sizes of microplastic particles, the analysis also examined glass microparticles, which showed significantly higher levels of bacteria on small and large polyethylene particles than on glass.

A bacterial biofilm is formed by the large- or single-layer growth of microorganisms, which are arranged in orderly as well as complex structures composed of bacterial microcolonies. In the case of adhesion itself, extracellular polymers produced by microorganisms, liposaccharides (LPS), their cell wall proteins and extracellular structures, such as fimbriae or cilia, play a special role [64,68,69,70,71]. Fimbriae are tubular cytoplasmic outgrowths a few micrometers long. Bacterial cells equipped with these structures more easily overcome the repulsive forces between the negatively charged cell and the structure on which adhesion is to occur. Cilia, on the other hand, are associated with the motility of bacteria, making it easier for them to reach the surface, and once on it, they enable the search for other microorganisms, aiming to form microcolonies [72,73].

**Figure 3:**
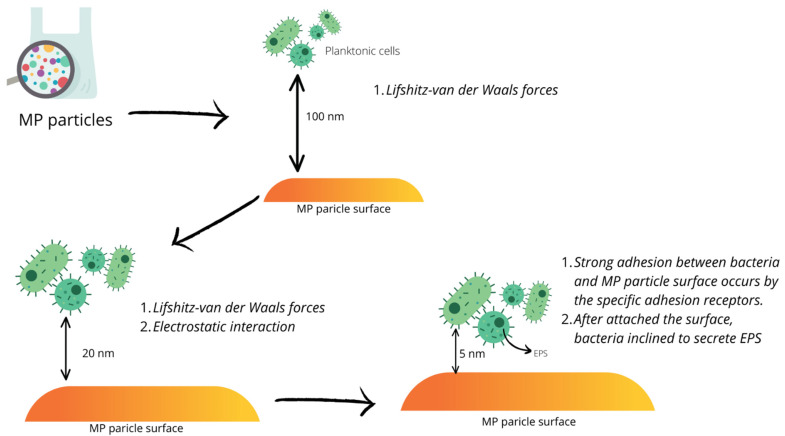
Simplified scheme of bacterial attachment to MPs (based on Wang et al. [74]).

In the initial phase of bacterial adhesion to the surface, Lifshitz–van der Waals forces and the electrostatic interaction between the cell surface and the substrate act indirectly and reversibly, which can lead to stronger adhesion via adhesion receptors. Subsequently, in an already irreversible stage of adhesion, extracellular polymers, LPS or proteins are produced, after which the microcolonies enlarge due to the proliferation of the first adherent cells [71,74]. Bacterial cells are connected through a polymeric substance (EPS) that consists of polysaccharides, proteins, nucleic acids, surfactants, lipids and water; however, the exact composition depends on the species of bacteria. EPS play important roles in biofilm formation and function, including the formation of a protective barrier that allows tolerance to bactericidal factors, avoidance of host-immune-system responses and predator attack, or bacterial cell aggregation, increased density and mutual recognition with transfer of genetic material (Figure 3). The supply of nutrients to the cells in the biofilm, on the other hand, is related to the formation of countless tubules in this structure and the fluid flowing in it, which envelops the microcolonies. This enables the delivery of essential nutrients and oxygen or the removal of unnecessary metabolic products. This system does not have access to all cells because most of the tubules are located at the periphery of the microcolonies. The partially cut off bacteria that do not have access to the tubules are, thus, in a state of anabiosis [73,75,76].

## 4. Microorganisms Detected on Microplastics

Given that MPs occur in different environments and vary in their physical and chemical properties, the composition of microorganisms present on their surface may differ significantly. The colonization of MP surfaces occurs within minutes to hours. MPs can provide not only a surface for microorganisms to colonize, but also a source of energy for them. Microorganisms can affect degradation rates, displacement and toxicity levels, but MPs can also be a vector for the spread of pathogens [77,78,79]. The main microorganisms colonizing the MP surface are bacteria, followed by diatoms, coccoliths, bryozoans and, in small numbers, dinoflagellates and barnacles [77].

In a study conducted by Kelly et al. [78] on three rivers in the United States, it was shown that there were significant differences in bacterial composition due to MP habitat. MPs collected from the Chicago River site had the highest proportion of taxa—Sinobacteraceae (10.66%), Burkholderiales (10.28%), Alphaproteobacteria (6.4%), *Sphingomonas* (4.15%)—while the range from 3 to 2% was represented by *Verrucomicrobia* (2.30%), Proteobacteria (2.76%) and Gammaproteobacteria (2.50%), and below 2% Opitutae (1.85%), Saprospispiraceae (0.99%), Rhizobiales (1.62%), *Novosphingobium* (0.89%), *Aquabacterium* (0.74%) and *Acinetobacter* (0.71%). The bacterial pattern was similar in the DuPage River, where the main bacterial genera were also Burkholderiales (13.87%), Sinobacteraceae (6.22%), Alphaproteobacteria (5.78%) and *Sphingomonas* (4.43%); and below the 3% level the occurrence of Proteobacteria (2.66%), Gammaproteobacteria (2.11%), Rhizobiales (1.18%), *Novosphingobium* (1.33%), Saprospiraceae (1.30%) and *Verrucomicrobia* (1.13%) was observed. In contrast, in the samples collected from Nippersink Creek, the main bacterial taxa were Burkholderiales (12.97%), Alphaproteobacteria (4.59%), Xanthobacteraceae (4.38%), Proteobacteria (4.25%), *Aquabacterium* (4.19%) and *Azospirillum* (4.17%), while Rhizobiales (2.92%), Acinetobacter (2.23%), Sphingomonadaceae (2.19%) and *Novosphingobium* (2.19%) were found in the range from 3 to 2%; the *Opitutae* class was not found and the Sinobacteraceae family was detected at the level of 0.01%.

In contrast, a study by Frére et al. [19] showed the occurrence of differences in the MP microbiome due to material type (PE: polyethylene; PP: polypropylene; PS: polystyrene) and sampling period from the bay of Brest (Brittany, France). The study showed that, despite the high percentage of operational taxonomic units (OTUs; 94 ± 4%), which was common among all plastic polymers, PE showed distinct bacterial clusters compared to PP and PS samples. In addition, when samples were collected at different times, a greater variation was shown in samples collected in December compared to October. MP samples and their microbiome from the October period were characterized by the presence of bacteria from Alphaproteobacteria (*Litoreibacter*, Rhodobacteraceae, Rhodobacteriales and Sphingoonadales) and Gammaproteobacteria (Alteromonadaceae, Alteromonodales, Moraxellaceae, Pseudomonadales and Vibrionales). Samples from December, on the other hand, were characterized by the presence of bacteria of Flavobacteria (*Tenacibaculum*), Alphaproteobacteria (*Litoreibacter*, Rhodobacteraceae and Rhodobacteriales) and Gammaproteobacteria (Alteromonadaceae, Alteromonodales, *Psychromonas*, Psychromonaceae, *Oleibacter*, Moraxellaceae, Pseudomonadales, Vibrionales, *Leucothrix*, Thiotrichaceae and Thiotrichales). Additionally, in a study by Jiang et al. [80] conducted in different parts of the Yangtze River (Lvsi, Chongning island and Xiangshan bay), variability was shown to be dependent on the prob collection site. In the Lvsi site, the MP microbiome was characterized mainly by the following families: Erythrobacteraceae, Rhodobacteraceae, Alteramonadaceae, Moraxellaceae and Oceanospirillaceae. At the Chongning island site, closer to the estuary, the MP microbiome consisted mainly of the following families: Gemmatimonadaceae, Sphingomonadaceae, Erythrobacteraceae, Alteramonadaceae, Lachbospiraceae and Comamonadaceae. On the other hand, in the East China Sea estuary area of Xiangsham bay, the main families were Erythrobacteraceae, Saprospiraceae, Chitynophagaceae, Acetobacteriaceae, Rhodobaqcteraceae and Cyanobacteria phylum.

The analysis of the microbial composition on the MP surface in the 2013 study by Zettler et al. [81] on the North Atlantic Subtropical Gyre showed that there were differences depending on the type of MP (PP or PE). Similar to the Frére et al. [19] study, bacteria present on PE had a higher diversity than on PP. The PE surface showed the occurrence of the following bacteria: Actinobacteria, Bacteroidetes, Sphingobacteriia, Chitinophagaceae, Cyanobacteria, Alphaproteobacteria (Rhodobacteriales and Rhodobacteraceae) and Gammaproteobacteria. On the other hand, the following were detected on PP: Actinobacteria, Alphaproteobacteria (Rickettsiales) and Gammaproteobacteria (Oceanospiralles). The McGivney et al. [79] study also showed that, depending on the type from MP (PE, PS and PP), the bacterial colonies formed on them differed in composition. PE had significantly higher levels of Proteobacteria, Sphingomonadaceae, *Novosphingobium* and Plantcomycetaceae compared to PS and PP. However, PS and PP had higher levels of Alphaproteobacteria, Bacteroidetes and Sphingobacteriales. As indicated in the above publications, the bacterial composition of the biofilm on the MP surface mainly consists of Bacteroidetes, Cyanobacteria, Proteobacteria, Alphaproteobacteria, Gammaproteobacteria and Acinetobacter taxa (Table 1). On the other hand, the microbiological composition of MPs is influenced by factors related to the aquatic environment, such as the type of reservoir or watercourse, geographical location and sampling period—briefly, all related to changes in the aquatic environment [19,78,80]. In addition, the types of microplastics are significant factors that also influence the microbial diversity of the MP biofilm. In the studies presented above, in most cases, the greatest microbial diversity was found in MPs consisting of PE [19,80].

The occurrence of pathogenic microorganisms on the surface of MP has also been observed with concern. The first study demonstrating the problem was presented by Maso et al. [82], where they detected the occurrence of potentially harmful *Ostreopsips* sp., *Coolia* sp. and *Alexandrium tylori* on MP surfaces. In a study by Kesy et al. [83] conducted in the Baltic Sea, they found that, in addition to Alphaproteobacteria (Sphingomonadaceae, Devosiaceae and Rhodobacteraceae) and Gammaproteobacteria (Alteromonadaceae and *Pseudomonas*), the pathogenic bacteria *Vibrio* spp. were also present. The authors also suggest that *Vibrio* spp. are generally early colonizers of surfaces. These bacteria were also detected in a previously reported study by Frére et al. [19].

## 5. Microplastics and Microbial Safety in Aquaculture

As evidenced from the previous sections, microplastics are a novel substrate for the formation of biological membranes, characterized by the distinctive community structure of the biofilm compared to the natural materials [84,85,86,87,88]. Due to their considerate fragmentation and physical properties (especially durability,) plastic particles also provide bacteria, including the above-mentioned pathogens, with an effective means of dispersion throughout the aquatic environment [86]. Van der Meulen et al. [87] in their report considering the risk assessment of “Socio-economic impact of microplastics in the 2 Seas, Channel and France Manche Region” pointed at a variety of potentially pathogenetic bacteria associated with marine litter, namely, *Bacteroides thetaiotaomicron*, *Escherichia coli*, *E. fergusoni*, *Shewanella putrefaciens*, *Bacillus cereus*, *B. thuringiensis*, *Aliivibrio wodani*, *Stenotrophomonas maltophilia* and *Pseudomonas anguilliseptica*. The matter is particularly significant for aquaculture, wherein interactions between humans (i.e., working staff and consumers) and farmed animals (catching, selection and consumption) are intensive. In this section, we present a review of the following: 1. etiological agents of cultured organisms’ diseases detected on plastic fragments, 2. human pathogens found on MP particles, 3. plant pathogens propagated by the pollutant, and 4. other plastic-associated issues affecting the health of aquacultural fauna.

### 5.1. Marine Animals’ Pathogens

The microbiological safety of animals farmed within aquaculture is an obvious issue. Diseased individuals will die before the harvest or achieve smaller weight gains. The market value of fish and sea cucumbers covered by ulcers and wounds will decrease, if they are released for sale at all. All this will cause direct financial losses, let alone the cost (financial and consumer health-related) of the antibiotic treatment. Here, we summarize the bacterial genera and species recorded to date on the plastic pollutants sampled from seas and freshwater reservoirs, plastic aquacultural equipment as well as on plastic materials intentionally submerged in waters to acquire biofilms for further examination. The results of in situ experiments performed in Sungo Bay, China, by Sun et al. [89] aimed at comparing bacterial fauna of mariculture-derived plastics (fishing nets, foams and floats) vs. natural feathers in the shellfish mariculture area and nearshore site and revealed the presence of bacterial genera potentially pathogenetic. Among them, *Vibrio* sp. showed the highest dominance of MPs, both in the mariculture area and near the shore. Similar observations were made by Zettler et al. [80] based on samples from the North Atlantic. In contrast, the *Vibrio* sp. detected by Jiang et al. [81] in the Yangtze River estuary, China, revealed the lowest prevalence in the local plastisphere. No matter how abundant, *Vibrio* species are the most numerous pathogens of farmed animals associated with MPs in the aquatic environment. *Vibrio parahaemolyticus* has been discovered in North and Baltic seas and *V. alginolyticus* in North Sea and in the lake Macquarie on PVC, PE and PP substrata. In the afore-mentioned lake *V. campbelli* was also present [90,91]. Both species are known to inhabit plastic particles collected from the mangrove ecosystem too [88]. *Vibrio parahaemolyticus*, along with other *Vibrio* spp., were found in skin ulcers of farmed Red Sea bream (*Pargus major*) and wild fish in Japan [92]. *Vibrio alginolyticus* is known to cause vibriosis in Pacific white shrimp (*Litopenaeus vannamei*), and together with *V. splendidus*, entailed larval and juvenile mortality in cultured carpet shell clams (*Ruditapes decussatus*) in 2001–2002, in Spain. The latter species is known to date to colonize plastic fragments in The Bay of Brest, France, together with *V. coralliilyticus* [19]. Clams from *Ruditapes* spp. are also prone to brown ring illness caused by *V. tapestis*. Diseased individuals are characterized by brown conchiolin deposits of variable distribution and thickness on the inner shell layers. Other farmed bivalves vulnerable to vibriosis include scallops: *Aequipecten irradians*, *Euvola ziczac*, *Argopecten purpuratus*, *Pecten maximus* and *A. ventricosus*; oysters: *Crassostrea virginica* and *C. gigas*; and abalone *Haliotis diversicolor supertexta* [93]. Four *Vibrio* species—*V. anguillarum*, *V. harveyi*, *V. pectenicida* and *V. xiamenensis*—were on a few plastic pieces in the Western Mediterranean Sea, as shown by Dussud et al. [94]. *Vibrio vulnificus*, in turn, recorded from Brazil is characterized by the serotype known to infect eels [95,96]. Some other representatives of *Vibrio* spp. are pathogenic towards farmed seahorses—i.e., *V. fortis*—related to enteritis in cultured *Hippocampus erectus* Perry, 1810. In holothuroids, the already mentioned *V. splendidus*, is also responsible for skin ulcerative syndrome (SUS) in edible Japanese spiky sea cucumber (*Apostichopus japonicus*, Holothuroidea). White shrimps are also affected by *V. harveyi* and *V. damsela* [97,98,99]. Aquaculture cage elements tested during the summer–autumn period in China revealed the presence of the above-mentioned *V. harveyi*, *V. vulnificus*, *V. splendidus* and, additionally, *V. fischeri* [100].

The presence in the plastisphere of undetermined *Vibrio* spp. in numerous of locations in China, along with France, Singapore, North and Baltic seas, confirms its leading role in the issue of pathogen vectoring by the discussed pollutant [19,81,83,101,102,103,104,105,106]. Moreover, Oberbeckmann and Labrenz [107] suggest that plastic particles are especially favorable means of transport for *Vibrio* spp. Additionally, *Vibrio*’s distinct affinity to the plastic surface is of particular importance in view of the prevalence of these bacteria in the marine environment in general. For example, *V. cholerae* was isolated at several locations in Chesapeake Bay in 1970s and, together with *V. parahaemolyticus* and related species, revealed a spatial and temporal distribution in an estuary [108]. Therefore, the detection of new plastic-inhabiting species and thus vectored by it is rather a matter of time and sample size. Finally, some peculiarities of animal farming conditions (i.e., crowding, higher temperature and a considerate amount of feces) provide especially favorable conditions for Vibrio, which can be subsequently propagated by MPs to ecologically sensitive areas [109].

Other pathogens detected on marine microplastics by Sun et al. [89] in China were *Alteromonas* spp., *Pseudoalteromonas* sp. and *Nautella* sp. (confirmed on microplastic also by Wang et al. [110]), the latter two including potentially opportunistic pathogens of the Pacific white shrimp. They are especially abundant in crustacean intestines after the exposure to ammonia and nitrite stress. Moreover, *P. piscicida* may cause high mortality in flower crab (*Portunus pelagicus*) [111,112,113]. *Pseudoalteromonas* spp. was also found on tested polypropylene, polystyrene and polyethylene fragments in the Bay of Brest, France, in Antarctica and aquacultural infrastructures in China [19,100,114].

Microplastics were a confirmed vector also of *Aeromonas salmonicida*, detected for the first time on plastic particles from North Adriatic Sea by Virsek et al. [86]. The bacterium is known to cause *Furunculosis salmonum*—a bacterial disease of cultured trout and salmon, cyprinids, pike, perch, bullheads, turbot and halibut. The genus was subsequently isolated from marine plastics from the West coast of Norway also by Radisic et al. [115]. Authors revealed the presence of three potentially virulent isolates of *A. salmonicida*. Prior to the above discoveries, Carballo et al. [116] experimentally tested the adhesion of *A. salmonicida* to materials used in aquaculture (polyvinyl chloride vs. polyethylene terephthalate vs. stainless steel) and revealed its greatest adhesion to plastics, in contrast to stainless steel. McCormick et al. [117] confirmed *Aeromonas* spp. and Campylobacteraceae family on freshwater plastics.

Another fish-related genus, *Tenacibaculum* spp., was confirmed on mariculture-associated MP biofilm in farming ponds and aquacultural equipment in China as well as in Western Mediterranean Sea [84,94,100]. These Gram-negative motile bacteria represent opportunistic pathogens for fish and can cause an ulcerative disease—*tenacibaculosis* (also known as salt “water columnaris” disease, a gliding bacterial disease of sea fish, bacterial stomatitis, eroded mouth syndrome and black patch necrosis). The condition is of considerable economic significance to aquaculture producers of many marine species. Diseased fish suffer from body lesions, gills and eyes necrosis, frayed fin, tail rot and eroded mouth. Affected individuals are prone to secondary infections from the open lesions [118]. *Tenacibaculum discolor* and *T. gallaicum* were isolated from sole (*Solea senegalensis*) and turbot (*Psetta maxima*) culture systems in China [100].

In the Western Mediterranean Sea, the crustacean and invertebrate pathogens *Phormidium* sp. and *Leptolyngbya* sp. were also observed and represented almost one third of the biofilm community [94]. The genus *Leptolyngbya*, whose members are the causative agents of the black band disease of corals, were also mentioned in research on plastisphere bacterial flora in the Chinese Xiangshan bay, together with *Pseudomonas* spp. by Jiang et al. [81]. The latter genus was also characterized by the relative abundance of 9%∼43.06% on the surface of plastic debris and 0.04%∼4.10% in the waters of Urumqi River, China [119]. A positive correlation between the plastic and potential pathogens was also confirmed by Zhang et al. [98] in the case of genera *Pseudomonas*, *Bacillus* and *Streptococcus*. Five species of *Pseudomanas* spp. were also detected by Curren and Leong [101], *P. alcaligenes*, *P. azotoformans*, *P. hussainii*, *P. pachastrellae* and *P. veronii*, in the tropical coastline.

Wang et al. [120] and Zhang et al. [105] revealed in the microplastic samples’ bacterial genera of *Muricauda*, *Ruegeria* and *Sunxiuqinia*.

### 5.2. Human Pathogens

The presence of human pathogens vectored by plastic particles in aquacultural farms is crucial for the health and safety of both the consumers and producers of aquacultural goods. The first group may be exposed to bacteria due to consumption, deliberately or not, of undercooked/raw seafood, while the second group is especially prone to tissue disruption while handling sharp-finned fish and crustaceans with tongs. The research conducted to date revealed pathogenic and opportunistic bacteria of humans on MP particles.

The best known among the hitherto-discussed human pathogenic bacterium is with no doubt *V. cholerae*—the etiological agent of numerous epidemics over the centuries worldwide. The species has been discovered on MP particles contained in the ballast waters of cruise ships and on plastic fragments in Brazil and was accompanied by *V. mimicus*—a species producing cholera toxins, able to cause gastroenteritis and being transmitted by raw oysters, fish, turtle eggs, prawns, squid and crayfish—and *V. vulnificus*, recently considered as a hazardous food-borne pathogen related to seafood, estimated at 50% mortality rate of developed infections. This microorganism can also infect wounds via contact with contaminated water or vibrio-vectoring shellfish [95,96,121,122,123]. Already mentioned *V. parahaemolyticus* along with *V. fluvialis* both found in the Baltic and North seas and *V. alginolyticus* (only in North Sea) [91] are other good examples of hazards for consumers. The importance of the first one results from the fact that the bacterium was originally known (in Japan, in 1950) as the etiologic agent of the human seafood-borne disease, causing high temperature and severe diarrhea. Recently, the illness has been especially abundant in Thailand; however, it can potentially occur worldwide [124,125]. *Vibrio fluvialis*, typically occurring in coastal habitats, is considered an emerging food-borne pathogen, causing diarrheal outbreaks and sporadic extraintestinal cases irrespectively of the economic conditions of the area [126]. Finally, *V. alginolyticus* is reported as etiologic agent (alone or in mixed flora) of human skin ulcers, otitis and ocular inflammation (mono-infection in professional fish cutters) [127]. It is also important for public health that *Vibrio* spp., along with *Escherichia coli*, have been detected in MPs deriving from the beach and Forth Estuary in Scotland [128,129]. The latter bacterium was also present in the plastisphere examined in Brazil by Silva et al. [96]. Experimental research on MP (collected from the intertidal sediment at coastal sites) pathogenic flora conducted in Argentina (Río de la Plata estuary) indicated that the biofilm was colonized bacteria, indicating fecal contamination (*E. coli* and Enterococci) [130]. Feces-associated bacteria (*E. coli* and *Enterococcus faecalis*) were also present in a Turkish dam lake (where MPs were present) and on a maricultural cage in China [100,131].

An opportunistic pathogen—*Moragnella morganii*—has been discovered in Norway, along with *Acinetobacter beijerinckii* [115]. The first one is a Gram-negative bacillus inhabiting the intestinal tract of humans, other mammals and reptiles. Confirmed bacteremia caused by the species included skin and soft tissue infections, pyelonephritis, female genitalia infections, pneumonia, gangrenous appendicitis and tonsillitis [132]. *Acinetobacter beijerinckii*, in turn, is also a Gram-negative coccobacillus described from samples originating from humans, equines and the environment (soil, water and hospitals). It was detected in the wound of a patient hospitalized in Sweden in 1980 [133]. The presence of representatives of the *A. baumanii* complex in Turkey were described by Tavşanoğlu et al. [131]. *Acinetobacter* sp. was also detected in plastic debris from the Urumqi River, China, by Xue et al. [111] in 2020. The authors revealed that bacterial abundance on artificial substratum was 0.01%∼12.09% vs. 0–0.1% in the waters around.

The genus *Arcobacter*, including human and animal pathogens, was recorded from microplastics originating from the Humber Estuary, UK, by Harrison et al. [134].

The wound-infecting *Aeromonas veronii* was detected on the already-mentioned aquacultural equipment elements in China by Hou et al. [100].

Wu et al. [85] experimentally compared the bacterial fauna of polyvinyl chloride (PVC) microplastic pellets vs. leaves and quartz particles incubated in water sampled from the Haihe River, China. The authors detected, among others, the presence of two opportunistic human pathogens occurring only on MPs: *Pseudomonas monteilii*, the causative agent of an exacerbation of bronchiectasis, also found in hospitals as an environmental contaminant [135], and *P. mendocina*, known to date from 14 cases of human bacteremia, meningitis, spondylodiscitis, endocarditis, foot wound infection and peritonitis [136]. The presence of *P. alcaligenes*, rarely opportunistic to humans, in turn, characterized plastic fragments deriving from coastline regions of Singapore [101]. *Pseudomonas* spp. have been detected in the estuary of the Yangtze River, aquacultural waters and equipment in China (especially abundant in the summer) and the West Coast of Norway as well [81,100,102,115].

In the Haihe River Estuary, *Shewanella* sp. was found in 2019 by Li et al. [103]. The genus was recently mentioned in Tan et al.’s [88] research on microplastics and sediment from the mangrove and by Laganà et al. [114] who found it on a polystyrene piece from King George Island (Antarctica). Representatives of *Shewanella* spp. (Gram-negative motile bacilli) may cause ears, skin and soft tissue infections, with or without bacteremia [137].

### 5.3. Plant Pathogens

This group comprises only one species to date; however, its relevance cannot be underrated, though the pathogen is not strictly related to aquaculture but rather agriculture. Wu et al. [85] discovered *P. syringae* in microplastic biofilms (in its absence, in quartz and leaf substrates) during the already-described experiment. According to Mansfield et al. [138], the bacterium ranks first on the list of the most dangerous plant pathogens. Therefore, in many countries of the world, it has the status of a quarantine organism.

### 5.4. Other Aspects of Microplastic Influence on Animal Health

Pathogen-enriched biofilms take on a special meaning in the context of reports pointing at increasing ingestion of, especially aged, plastic particles covered in bacterial films by marine copepods and amphipods. The latter group is a popular bait for cultured organisms. *Orchestia gammarellus* was revealed to ingest and subsequently shred a four-fold greater amount of plastic with biofilm than clean plastic, with the plastic type playing no role [139,140]. Fabra et al. [141] proved that plastic particles uptake by European native oysters (*Ostrea edulis*) was significantly higher when particles were covered by bacterial biofilms. According to Wang et al. [98], the biofilm-covered plastics emit olfactory signals promoting ingestion. This problem is related to the circulation and accumulation of plastics in trophic chains, resulting in the presence of the substance in a variety of seafoods [142], but also to a problem of the intestinal microbiome of the animals.

The above issue has been raised, among others, by Li et al. [143], Lu et al. [144], Wang et al. [98] and Fackelmann and Sommer [145], who pointed that ingested MP particles can scratch the gut walls causing inflammation and malnutrition, which combined with potential pathogens or opportunistic bacteria will result in a reduction in the intestine flora, leading to dysbiosis in chronically exposed organisms, which will eventually affect the immune system. The detrimental effect of ingested plastic on aquacultural organisms’ health was observed in mussels, oysters, shrimps and red tilapia [146,147,148,149,150,151].

Finally, farmed animals may also suffer from harmful algal blooms and mucilage events (caused by dinoflagellates *Ostreopsis*, *Coolia* and *Alexandrium taylori* and diatoms *Ceratoneis closterium*, *Coscinodiscophytina* and *Bacillariophytina*) vectored on plastic particles, which have been to date detected in ballast waters as well as in the North and Mediterranean seas [104,122,151].

### 5.5. Perspectives

**Figure 4:**
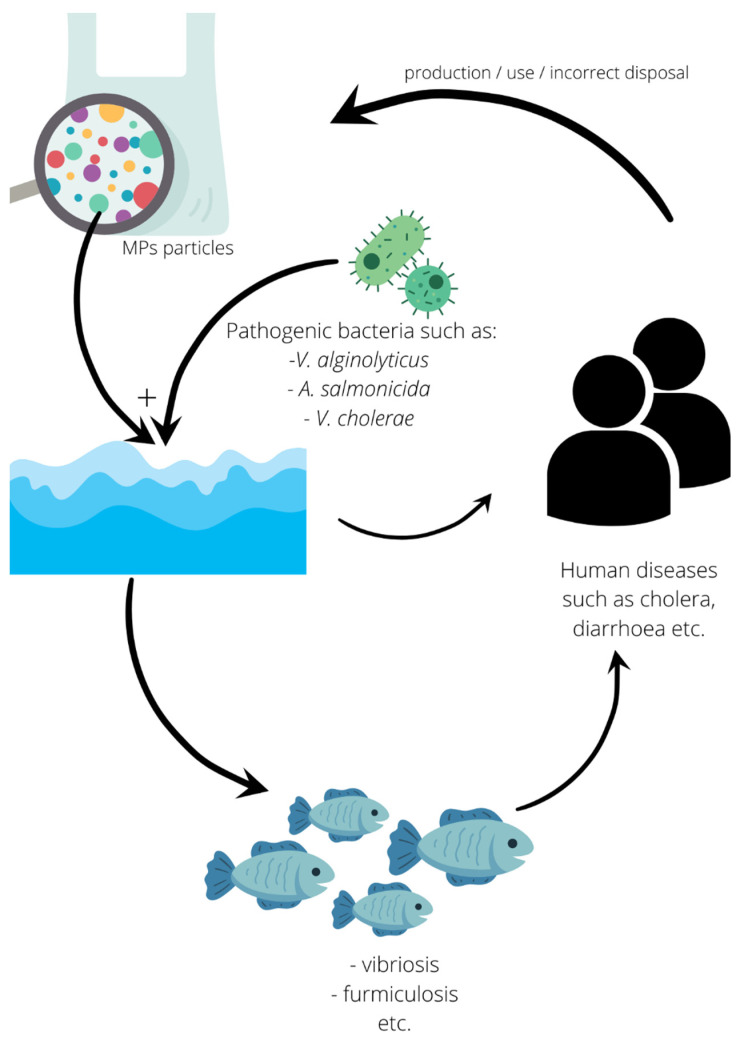
Microplastic as a vector of pathogens [19,86,95,96,121,122,123,124,125].

The problem of plastic contamination needs an urgent solution, not solely for the sake of aquaculture. Managing it seems to fit in with the idea of one health (Figure 4). as “an integrated, unifying approach that aims to sustainably balance and optimize the health of people, animals, and ecosystems” (after: Joint Tripartite (FAO, OIE, WHO) and UNEP Statement, December 2021). Additionally, in our view, only the recognition and acknowledgment of the close links and interdependence between the health of the environment, wild and domestic animals as well as humans can lead to sustainable development. This is why stopping the microplastic flood requires the synergistic effort of industrial and social parties, resulting in the already commonly understood reduction in and/or replacement of this material by healthier substitutes, and thus starting the slow process of the drug-free purification of the food chains, at the top of which stands the human species.

## 6. Conclusions

The above summarized the current state of our knowledge regarding the implications of plastic presence in aquatic environments, which is not optimistic for the future. This ubiquitous pollutant, inseparable from human activity, affects micro- and macro-organisms, tending to accumulate in the food chain, resulting in the intoxication of the animals, mechanical damage to their internal organs along with microbiome disturbance. Furthermore, the listed threats are strictly related to the considerable affinity shown by a variety of bacteria to the MP surface. Biofilms are easily created on different plastic types and its presence is known to facilitate the ingestion of the material by organisms that are lower links of the trophic chain and are frequently used as a food source for farmed fish. The latter, combined with the fact that many pathogenic bacteria are more abundant (including drug-resistant strains) on plastic particles than on natural substrates, should be paid particular attention. Plastic’s durability and mobility make it a perfect vector for the pathogens discussed in this review, and its victims may become the organisms kept in closed systems, consumers of aquatic foods and aquacultural staff.

## Figures and Tables

**Table 1:** Selected microorganisms present on the MP surface (>5 mm).

Group	Selected Taxa	Reference
**Bacteria**	Sinobacteraceae, Burkholderiales, Alphaproteobacteria, *Sphingomonas*, *Verrucomicrobia*, Gammaproteobacteria, Opitutae, Saprospispiraceae, Rhizobiales, *Novosphingobium*, *Aquabacterium*, *Acinetobacter*, Xanthobacteraceae, *Azospirillum*, *Acinetobacter*, Sinobacteraceae	[77,78,80]
**Diatoms**	*Amphora*, *Achanathes*, *Cymbella*, *Grammatophora*, *Haslea*, *Licophora*, *Microtabella*, *Minidiscus*, *Thalassionema*, *Thalassiosira*, *Chaetoceros*, *Mastogloia*, *Navicula*, *Nitzschia*, *Sellaphora*, *Strauroneis*	[80]
**Coccoliths**	*Calcidiscus*, *Emiliania*, *Gephyrocapsa*, *Umbellospharea*, *Umbilicospharea*, *Coccolithus*, *Calciosolenia*	[77]
**Insect eggs**	*Halobates*	[77]
**Barnacles**	*Lepas*	[77]
**Dinoflagellates**	*Alexandrium*, *Ceratium*	[80]
**Ciliates**	*Ephelota*	[80]

## Data Availability

Not applicable.
